# Perinatal obsessive-compulsive disorder in fathers

**DOI:** 10.1192/j.eurpsy.2023.1960

**Published:** 2023-07-19

**Authors:** I. Simões, G. Andrade, B. Côrte-Real, A. Duarte, D. Pereira

**Affiliations:** Psychiatry and Mental Health, Santa Maria Hospital, Centro Hospitalar Universitário Lisboa Norte EPE, Lisboa, Portugal

## Abstract

**Introduction:**

Obsessive–Compulsive Disorder (OCD) is a disabling and chronic illness defined by the presence of obsessions and/or compulsions. Recently it has been proposed that the perinatal period may act as a trigger in this disorder, leading to its onset or exacerbation.

**Objectives:**

Variations in pregnancy-related hormones are believed to be one of the main etiological theories for the development of perinatal OCD (pOCD). Perhaps for that reason research has been almost exclusively focused on the development of this disorder in mothers. We aim to investigate pOCD in fathers.

**Methods:**

A non-systematic review was conducted via electronic searches of PubMed. The keywords used were “Perinatal”, “Father”, “OCD”, “Obsessive-compulsive disorder”.

**Results:**

Unwanted intrusive thoughts are experienced with a similar prevalence in mothers and fathers. The same seems to be true regarding compulsions. However, it does appear that mothers are more distressed by these symptoms, which tend to be baby-related, usually concerning themes of suffocation, accidents or contamination. It is hypothesized that this seemingly different impact is related to the fact that mothers are more often the primary caregivers than fathers, thus feeling more distress because they are imbued with a greater responsibility. Accordingly, pOCD symptoms tend to be more severe in fathers who consider their baby-related obsessions meaningful, often confusing them as a desire to carry out such thoughts. These findings are consistent with the Cognitive-Behavioral Theory of OCD, highlighting that purely biological theories for the development of pOCD might not suffice.

**Conclusions:**

Research indicates a similar presence of OCD symptoms in postnatal mothers and fathers, although it seems that mothers may experience more distress. Underlying dysfunctional beliefs seem to be responsible for the negative appraisal of these symptoms, predicting the development of the disorder in question. Further research of pOCD should seek to better characterize the onset or exacerbation of this disorder in fathers.

**Disclosure of Interest:**

None Declared

